# Sustained Pericarditis Recurrence Risk Reduction With Long‐Term Rilonacept

**DOI:** 10.1161/JAHA.123.032516

**Published:** 2024-03-12

**Authors:** Massimo Imazio, Allan L. Klein, Antonio Brucato, Antonio Abbate, Michael Arad, Paul C. Cremer, Antonella Insalaco, Martin M. LeWinter, Basil S. Lewis, David Lin, Sushil A. Luis, Stephen J. Nicholls, Paul Sutej, Yishay Wasserstrum, JoAnn Clair, Indra Agarwal, Sheldon Wang, John F. Paolini, for the RHAPSODY Investigators

**Affiliations:** ^1^ Department of Medicine (DMED), University of Udine and Cardiothoracic Department University Hospital Santa Maria della Misericordia, ASUFC Udine Italy; ^2^ Cleveland Clinic Cleveland OH USA; ^3^ University of Milano, Fatebenefratelli Hospital Milan Italy; ^4^ Berne Cardiovascular Research Center, School of Medicine University of Virginia Charlottesville VA USA; ^5^ Leviev Heart Center, Ramat Gan Tel Aviv University School of Medicine Ramat Gan Israel; ^6^ Division of Rheumatology, Ospedale Pediatrico Bambino Gesù IRCCS [European Reference Network (ERN) for Rare Immunodeficiency, Autoinflammatory and Autoimmune Diseases (RITA) Center] Rome Italy; ^7^ University of Vermont Medical Center Burlington VT USA; ^8^ Lady Davis Carmel Medical Center and Technion‐Israel Institute of Technology Haifa Israel; ^9^ Abbott Northwestern Hospital Minneapolis MN USA; ^10^ Mayo Clinic Rochester MN USA; ^11^ Victorian Heart Institute Monash University Clayton Australia; ^12^ Northside Hospital Atlanta GA USA; ^13^ Kiniksa Pharmaceuticals Lexington MA USA

**Keywords:** autoinflammatory disease, interleukin‐1, recurrent pericarditis, rilonacept, Pericardial Disease

## Abstract

**Background:**

Rilonacept, a once‐weekly interleukin‐1 alpha and beta cytokine trap, reduced pericarditis recurrence in the phase 3 study, RHAPSODY (Rilonacept Inhibition of Interleukin‐1 Alpha and Beta for Recurrent Pericarditis: A Pivotal Symptomatology and Outcomes Study). The RHAPSODY long‐term extension further explored recurrent pericarditis natural history and treatment duration decision‐making during 24 additional months of open‐label rilonacept treatment.

**Methods and Results:**

Seventy‐four patients commenced the long‐term extension, with a median (maximum) total rilonacept duration of 22 (35) months. Individually, 18 months after the most proximal pericarditis recurrence, investigators decided to continue rilonacept on study, suspend rilonacept for off‐treatment observation (rescue allowed), or discontinue the study. The annualized incidence of pericarditis recurrence on rilonacept up to the 18‐month decision milestone was 0.04 events/patient‐year versus 4.4 events/patient‐year prestudy while on oral therapies. At the 18‐month decision milestone, 64% (33/52) continued rilonacept, 15% (8/52) suspended rilonacept for observation, and 21% (11/52) discontinued the study. Among the 33 patients (1/33; 3.0%) continuing rilonacept (median time to recurrence could not be estimated due to too few events), a single recurrence occurred 4 weeks after a treatment interruption. Among patients suspending rilonacept, 75% (6/8) experienced recurrence (median time to recurrence, 11.8 weeks [95% CI, 3.7 weeks to not estimable]). There was a 98% reduction in risk of pericarditis recurrence among patients continuing rilonacept treatment after the 18‐month decision milestone versus those suspending treatment for observation (hazard ratio, 0.02; *P*<0.0001).

**Conclusions:**

In the RHAPSODY long‐term extension, continued rilonacept treatment resulted in continued response; treatment suspension at the 18‐month decision milestone was associated with pericarditis recurrence.

**Registration:**

URL: https://www.clinicaltrials.gov; Unique identifier: NCT03737110.

**Nonstandard Abbreviations and Acronyms**
LGE: late gadolinium enhancement
NRS: numerical rating scale
RHAPSODY: Rilonacept Inhibition of Interleukin‐1 Alpha and Beta for Recurrent Pericarditis: A Pivotal Symptomatology and Outcomes Study

**Clinical Perspective**

**What Is New?**

- In RHAPSODY (Rilonacept Inhibition of Interleukin‐1 Alpha and Beta for Recurrent Pericarditis: A Pivotal Symptomatology and Outcomes Study), continued rilonacept treatment for a median of 2 years during the long‐term extension resulted in continued treatment response.
- Rilonacept reduced the risk of pericarditis recurrence by 98% beyond 18 months of treatment.

**What Are the Clinical Implications?**

- In patients with recurrent pericarditis, consistent treatment for the full duration of the disease without interruption may be warranted for long‐term pericarditis recurrence prevention.

Recurrent pericarditis is a painful and debilitating chronic disease (median disease duration, 2.84 years)^1^, ^2^, ^3^ with detrimental impacts on quality of life.^1^, ^2^, ^3^, ^4^, ^5^, ^6^, ^7^

Interleukin‐1, a key mediator in recurrent pericarditis, is an ideal target for specific intervention in patients with systemic inflammation (eg, elevated CRP [C‐reactive protein]).^4^, ^5^ Rilonacept is a once‐weekly interleukin‐1 alpha and beta cytokine trap that prevents engagement with the cell‐surface receptor, inhibiting both interleukin‐1 alpha and beta activity.^8^

The efficacy and safety of rilonacept in recurrent pericarditis were demonstrated in the pivotal placebo‐controlled phase 3 trial RHAPSODY (Rilonacept Inhibition of Interleukin‐1 Alpha and Beta for Recurrent Pericarditis: A Pivotal Symptomatology and Outcomes Study).^4^ During an event‐driven randomized‐withdrawal period (median, 9 months), rilonacept treatment reduced pericarditis recurrence risk by 96% (hazard ratio [HR], 0.04 [95% CI, 0.01–0.18]; *P*<0.001). Median time to recurrence could not be estimated in the rilonacept group and was 8.6 weeks (95% CI, 4.0–11.7) in the placebo group. The 2 recurrence events in the rilonacept group (annualized incidence, 0.15 events/patient‐year)^4^, ^9^ were associated with temporary interruptions of 1 to 3 weekly doses.

The RHAPSODY open‐label extension was designed to elicit further insights into long‐term rilonacept efficacy and safety and to inform clinical decision‐making and the natural history of the disease.

## METHODS

### Study Design and Patients

RHAPSODY (ClinicalTrials.gov, NCT03737110), a phase 3, double‐blind, placebo‐controlled, event‐driven, randomized‐withdrawal trial of rilonacept in patients with recurrent pericarditis, included a prespecified long‐term extension of up to 24 months additional open‐label rilonacept treatment.^4^ RHAPSODY was conducted in accordance with the principles of the Declaration of Helsinki, the Good Clinical Practice guidelines of the International Council for Harmonisation of Technical Requirements for Pharmaceuticals for Human Use, and all relevant regulations. The protocol was approved by the relevant institutional review boards or independent ethics committees for all participating centers. All patients provided written informed consent. Data supporting the findings of this study are available upon reasonable request.

RHAPSODY design and pivotal study results have been published.^4^, ^8^ Briefly, eligible adults and children 12 years or older with recurrent pericarditis presented with acute signs and symptoms that constituted at least a second recurrence despite treatment with nonsteroidal anti‐inflammatory drugs (NSAIDs), colchicine, and/or oral glucocorticoids; scored ≥4 on the 11‐point (0 [none] to 10 [greatest severity]) numerical rating scale (NRS) for pain^10^; and had CRP of at least 1 mg/dL within 7 days of first study drug administration.

**Figure 1:**
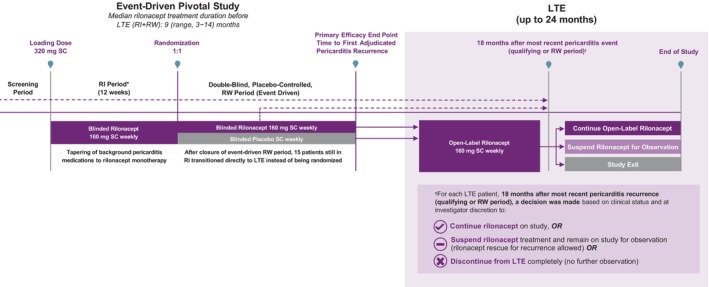
RHAPSODY design. Patients who completed the randomized‐withdrawal period while on study drug and were clinically stable were eligible to continue treatment with rilonacept (160 mg subcutaneously self‐ or caregiver‐administered once weekly) in the open‐label, long‐term extension. Patients still in the run‐in when the event‐driven randomized‐withdrawal period ended and who had achieved the defined clinical‐response criteria were transitioned directly into the long‐term extension. *Duration of the RI period was concealed from patients to blind them to randomization timing. LTE indicates long‐term extension; RHAPSODY, Rilonacept Inhibition of Interleukin‐1 Alpha and Beta for Recurrent Pericarditis: A Pivotal Symptomatology and Outcomes Study; RI, run‐in; RW, randomized‐withdrawal; and SC, subcutaneous.

Over a 12‐week run‐in, all patients received rilonacept during weaning from background pericarditis medications (Figure 1). Patients meeting prespecified clinical‐response criteria (including no pericarditis recurrence) at the end of run‐in were randomized to weekly rilonacept or placebo in the event‐driven randomized‐withdrawal period, which closed when a prespecified number of adjudicated pericarditis events had accrued.

Clinically stable patients completing the randomized‐withdrawal period were eligible to continue into the open‐label, long‐term extension and receive weekly rilonacept. Patients still in the run‐in when the event‐driven randomized‐withdrawal period ended and who had achieved the defined clinical‐response criteria entered the long‐term extension directly. Concomitant oral pericarditis medications (except steroids) were allowed, unlike the randomized‐withdrawal period, and patient participation ended after 24 months of treatment, upon treatment discontinuation, or rilonacept US commercial launch (US patients only).

Individually, 18 months after the most recent pericarditis recurrence (qualifying episode or randomized‐withdrawal period flare), based on clinical status and investigator discretion (18‐month decision milestone), patients continued rilonacept treatment on study, suspended rilonacept treatment but remained on study for observation off treatment (rescue/bailout allowed), or discontinued the study without further observation other than a 6‐week safety follow‐up (no option for rescue/bailout). This terminology for the 18‐month decision milestone was intentionally chosen to distinguish among the different management strategies. Long‐term extension recurrences were investigator assessed, different from the independent adjudication for the randomized‐withdrawal period; however, individual clinical outcomes measures (ie, pain, CRP, ECG, presence/absence of effusion by echocardiogram, and presence/absence of pericardial friction rub) were collected and were available for post hoc review. Bailout rilonacept was available for administration for pericarditis recurrences while on study.

### Outcomes Measures

Efficacy outcomes included time to pericarditis recurrence after the 18‐month decision milestone and annualized recurrence rate before and after the 18‐month decision milestone. Secondary outcomes included Patient Global Impression of Pericarditis Severity (PGIPS) and Physician Global Assessment of Pericarditis Activity (PGA‐PA) (Data S1). CRP levels were measured at baseline and every 12 weeks. Patients assessed pain intensity using the pericarditis pain NRS every 12 weeks.

Safety outcomes included treatment‐emergent adverse events, that is, those starting or increasing in severity from long‐term extension start through 6 weeks after final rilonacept administration. The data monitoring committee reviewed safety data throughout the duration of the study.

RHAPSODY run‐in and randomized‐withdrawal period data updates resulting from the final database lock and closing of the long‐term extension are provided in Table S1 and Table S2.

### Data Analysis

Time to pericarditis recurrence after the 18‐month decision milestone was defined as the time to the date of pericarditis recurrence, either censored at end of study or at last assessment before treatment resumed (if rilonacept was restarted in the off‐treatment period in the absence of recurrence), whichever came first.

### Statistical Analysis

Descriptive statistics were used to report efficacy outcomes of time to pericarditis recurrence and annualized recurrence rates. The prespecified time to recurrence analysis used a Cox proportional‐hazards model to estimate the HR and was quantified with a log‐rank test by providing a nominal *P* value for comparing treatment continuation versus off‐treatment observation. The Kaplan–Meier method was used to estimate survival functions. Methods for annualized recurrence rate calculation are provided in Data S1.

## RESULTS

### Patient Disposition

**Figure 2:**
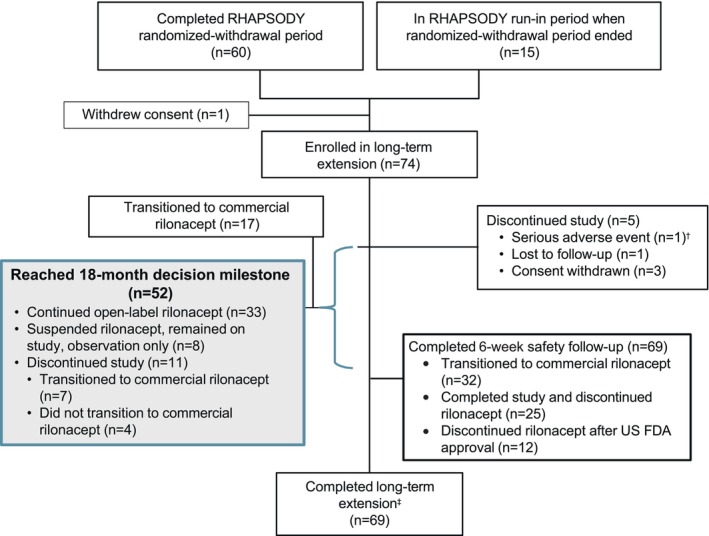
Patient disposition during long‐term extension of phase 3 RHAPSODY.* Of the 74 eligible patients who were enrolled in the open‐label, long‐term extension of RHAPSODY, 69 patients completed this phase of the study. *Patient disposition data for RHAPSODY study periods before long‐term extension have been published. ^†^Investigator decision to discontinue patient due to serious adverse events of acute endocarditis. ^‡^44 US patients either transitioned to rilonacept after US Food and Drug Administration approval (n=32; completed subsequent 6‐week safety follow‐up) or discontinued treatment (n=12; completed subsequent 6‐week safety follow‐up); 25 non‐US (Australia, Israel, Italy) patients remained on open‐label rilonacept until long‐term extension closure (completed subsequent 6‐week safety follow‐up). FDA indicates Food and Drug Administration; and RHAPSODY, Rilonacept Inhibition of Interleukin‐1 Alpha and Beta for Recurrent Pericarditis: A Pivotal Symptomatology and Outcomes Study.

Mean prestudy (pre‐run‐in) disease duration was 2.5 years (annualized incidence, 3.7 events/patient‐year); median treatment duration (preextension) was 9 months. In May 2020, 74 of 75 eligible RHAPSODY patients continued into open‐label long‐term extension (Figure 2). Mean age was 44.2 years; 54.1% were female (Table 1). The predominant underlying cause was idiopathic (82.4%), with 16.2% due to postcardiac injury.

**Table 1:** Baseline Demographics and Clinical Characteristics of Long‐Term Extension Study Cohort at Run‐In*

Characteristic	N=74
Age, y	44.2±15.6
Age, y, distribution, no. (%)	
12–17	6 (8.1)
18–64	62 (83.8)
65–78	6 (8.1)
Sex, no. (%)	
Male	34 (45.9)
Female	40 (54.1)
Race, no. (%)	
White	68 (91.9)
Black	5 (6.8)
Other	1 (1.4)
Geography, no. (%)	
United States	45 (60.8)
Non‐US (Italy, Israel, Australia)	29 (39.2)
Recurrent pericarditis type, no. (%)	
Idiopathic	61 (82.4)
Postpericardiotomy syndrome	12 (16.2)
Dressler syndrome	1 (1.4)
Medication used at qualifying episode of pericarditis, no. (%)	
NSAID	51 (68.9)
Colchicine	60 (81.1)
Corticosteroid	38 (51.4)
Disease duration, y	2.5±3.2
Total number of episodes of pericarditis, including index and qualifying episodes	4.8±1.7
Annualized incidence of pericarditis episodes	3.7±3.0
C‐reactive protein level (qualifying episode), mg/dL	6.4±6.9

* Plus–minus values are means ± SDs.

Median (maximum) rilonacept treatment duration from run‐in baseline to end of extension was 22 (35) months. The long‐term extension at US sites concluded in April 2021 after US approval of rilonacept for treatment of recurrent pericarditis: all 45 US patients either switched to commercial rilonacept therapy (n=32) or discontinued rilonacept (n=13); median (maximum) duration of rilonacept treatment from run‐in baseline was 18 (27) months. Among non‐US patients, median (maximum) duration of rilonacept treatment from run‐in baseline through study closure (June 2022) was 28 (35) months (see Consolidated Standards of Reporting Trials diagram; Figure 2).

### Efficacy Before 18‐Month Decision Milestone

The annualized incidence of pericarditis recurrence while on rilonacept treatment up to the 18‐month decision milestone was 0.04 events/patient‐year. In 3 investigator‐assessed pericarditis recurrences, pain NRS scores were 4 or higher, but CRP remained below the RHAPSODY event adjudication criterion threshold of 1 mg/dL; 1 patient had a new pericardial effusion. Two recurrences were managed with brief oral regimens (NSAID with/without colchicine); one patient received no rescue medication (Data S1). The proportion of patients with CRP levels at/below 0.5 mg/dL or at/below 1 mg/dL at each study visit ranged from 91.4% to 100% and 95.5% to 100%, respectively.

During the randomized‐withdrawal period, all patients received only rilonacept monotherapy. During the extension before the 18‐month decision milestone, only 6 patients added colchicine (for 2–9 months), all without reported recurrence (5 prophylactically, 1 for chest pain).

### Efficacy After 18‐Month Decision Milestone

Fifty‐two patients (70.3%) reached the 18‐month decision milestone (ie, 18 months after most proximal recurrence— the qualifying episode [n=36] or randomized‐withdrawal recurrence [n=16]) (Figure 2). At the visit, 92.2% (47/51) of patients reported absent/minimal pericarditis symptoms (PGIPS score, 0 or 1), and 94.2% (49/52) had absent/minimal pericarditis activity (PGA‐PA ratings). All had CRP levels <1 mg/dL, absent pericardial rub, normal electrocardiogram, and no pericardial effusion. On the day of the 18‐month decision milestone, 28 patients had cardiac magnetic resonance (CMR) imaging scans, of whom 3 patients had moderate late gadolinium enhancement (LGE), 2 patients had mild LGE, and 23 had none/trace/not measurable LGE. Subsequently, 33 (63.4%) continued rilonacept treatment (14 CMRs at 18‐month decision milestone: 1 moderate LGE, 1 mild LGE, 12 none/not measurable); 8 (15.4%) suspended rilonacept (without taper) (7 CMRs at 18‐month decision milestone: all none/trace LGE), remaining on study for observation off treatment; and 11 (21.2%) discontinued study participation (7 CMRs available at 18‐month decision milestone: 2 moderate LGE, 1 mild LGE, 4 none/trace LGE) of whom 7 transitioned to commercial rilonacept. Patients discontinuing study participation without transition to commercial rilonacept stopped on‐study rilonacept without taper and received a 6‐week safety follow‐up.

**Figure 3:**
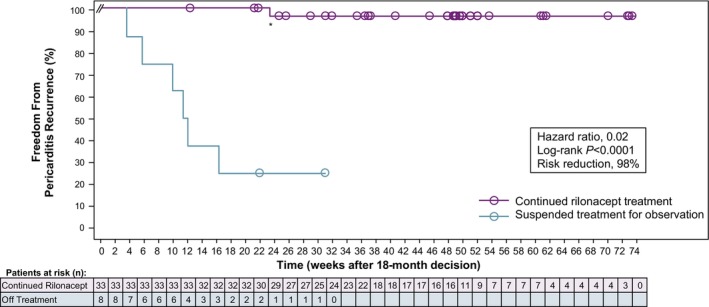
Treatment response after 18‐month decision milestone. Pericarditis recurrence risk was reduced among patients who continued rilonacept treatment after the 18‐month decision milestone compared with patients who suspended treatment and remained under observation (rescue allowed) (hazard ratio, 0.02; *P*<0.0001). *The patient with a recurrence at 23.4 weeks had interrupted rilonacept treatment approximately 4 weeks prior for elective cardiac surgery.

There was a 98% reduction in pericarditis recurrence risk among patients continuing rilonacept treatment after the 18‐month decision milestone (n=33) versus those suspending treatment for observation (n=8) (HR, 0.02; *P*<0.0001) (Figure 3). Among the 33 patients continuing rilonacept, 1 (recurrence rate, 3.0%) had an investigator‐assessed recurrence (NRS 8 and CRP 7.5 mg/dL, meeting the RHAPSODY event adjudication criteria) 23.4 weeks into the long‐term extension, associated with an intentional 4‐week rilonacept treatment interruption 2 weeks before elective surgery. Rilonacept was restarted after a failed attempt of NSAID/colchicine rescue therapy.

Among the 8 patients who suspended rilonacept but remained under observation, 6 had an investigator‐assessed recurrence (recurrence rate, 75%) with NRS >7 and CRP >1 mg/dL (ie, meeting the RHAPSODY event adjudication criteria). Median (interquartile range) time to recurrence after suspending rilonacept was 11.8 (3.7 to not estimable) weeks. In 4 cases, attempted rescue with combination NSAID/colchicine failed. Reinitiation of rilonacept treatment (n=5) resulted in resolution of pericarditis recurrences; no additional recurrences were observed. Three patients received prophylactic colchicine for at least 3 months, of whom 2 had a recurrence and 1 did not (see recurrence details in Data S1). Annualized recurrence rates were 0.18 (95% CI, 0.06–0.41) events/patient‐year for those continuing rilonacept and 2.18 (95% CI, 0.80–4.75) events/patient‐year for those suspending rilonacept.

During the posttreatment 6‐week safety follow‐up after discontinuing rilonacept study drug (n=25 non‐US patients after study closure; n=12 US patients completing the study without transition to commercial rilonacept), 5 additional investigator‐assessed pericarditis recurrences (NRS >6; CRP >1 mg/dL; met RHAPSODY adjudication criteria) were reported, at 3 weeks (in 1 patient) and at 6 weeks (in 4 patients) (see Data S1). Only 3 patients started prophylactic colchicine at the time of discontinuing rilonacept, none of whom had a reported recurrence.

### Safety

In total, 83.8% (62/74) of patients experienced treatment‐emergent adverse events (Table 2). Maximum severity was mild (36.5%) or moderate (37.8%), with no deaths and no malignancies. Of 6 serious adverse events, 2 (acute endocarditis, viral pneumonia) were considered possibly study‐drug related. Adverse events of COVID‐19 and sinus congestion led to treatment interruption; fatigue, acute endocarditis, and viral pneumonia led to study drug discontinuation. The most common adverse event (16.2%, 12 patients) was upper respiratory tract infection. Injection‐site reactions were reported in 5 patients (6.8%).

**Table 2:** Adverse Events in RHAPSODY*

Category†, no. (%)	Run‐in (N=86)	Randomized‐withdrawal period				Long‐term extension (N=74)
		Rilonacept, including bailout (N=30)	Placebo, including bailout (N=31)	Rilonacept before bailout (N=30)	Placebo before bailout (N=31)	
Any adverse event‡	70 (81.4)	25 (83.3)	23 (74.2)	25 (83.3)	13 (41.9)	62 (83.8)
Adverse events by maximum severity§						
Mild	53 (61.1)	17 (56.7)	18 (58.1)	17 (56.7)	9 (29.0)	27 (36.5)
Moderate	15 (17.4)	8 (26.7)	4 (12.9)	8 (26.7)	3 (9.7)	28 (37.8)
Severe	2 (2.3)	0	1 (3.2)	0	1 (3.2)	7 (9.5)
Adverse events related to study drug‖	47 (54.7)	11 (36.7)	5 (16.1)	11 (36.7)	1 (3.2)	22 (29.7)
Serious adverse events¶	1 (1.2)	1 (3.3)	3 (9.7)	1 (3.3)	1 (3.2)	6 (8.1)
Serious adverse events related to study drug#	0	0	0	0	0	2 (2.7)
Adverse events leading to dose interruption**	0	1 (3.3)	0	1 (3.3)	0	2 (2.7)
Adverse events leading to study drug discontinuation††	4 (4.7)	0	0	0	0	3 (4.1)
Adverse events leading to death	0	0	0	0	0	0
Infections or infestations‡‡	14 (16.3)	12 (40.0)	7 (22.6)	12 (40.0)	3 (9.7)	31 (41.9)
Upper respiratory tract infections	12 (14.0)	7 (23.3)	2 (6.5)	7 (23.3)	0	12 (16.2)
Injection‐site reactions	28 (32.6)	7 (23.3)	2 (6.5)	6 (20.0)	0	5 (6.8)
Malignancies§§	0	1 (3.3)	0	1 (3.3)	0	0

* Summary of all adverse events from all periods analyzed at time of final database lock after study completion.

^†^ Patients with multiple events were counted once in same category.

^‡^ Adverse event that started or increased in severity from first study drug dose in long‐term extension to 6 weeks after last dose in long‐term extension.

^§^ Each patient represented according to maximum severity.

^‖^ Each event was related, possibly related, or missing, as assessed by investigator.

^¶^ Six patients experienced serious treatment‐emergent adverse events: (1) pneumothorax; (2) acute endocarditis, aortic valve disease, acute myocardial infarction, pericarditis; (3) transient ischemic attack, coronavirus infection; (4) worsening of aortic insufficiency; (5) pneumonia, pneumonia viral (COVID‐19); and (6) left ventricular failure, hip fracture, bile duct stone, cardiac device malfunction.

^#^ Events included acute endocarditis and viral pneumonia.

** Events included coronavirus infection and sinus congestion.

^††^ Events included fatigue, acute endocarditis, and viral pneumonia.

^‡‡^ No reported infestation adverse events.

^§§^ Excluding basal cell carcinoma of skin.

## DISCUSSION

The previously‐reported RHAPSODY pivotal results demonstrated the efficacy of rilonacept in the treatment of recurrent pericarditis in symptomatic patients failing standard of care and in prevention of recurrence over a median 9‐month treatment duration. The long‐term extension expanded treatment duration to a median of 2 years (maximum, 3 years). The annualized incidence of pericarditis recurrence up to the 18‐month decision milestone while on rilonacept treatment (0.04 episodes/patient‐year) was substantially lower than during the 2.5‐year prestudy period (3.7 episodes/patient‐year) while on conventional oral therapies. After the 18‐month decision milestone, 75% (6/8) of patients who “suspended” rilonacept therapy experienced a recurrence (met RHAPSODY adjudication criteria of NRS ≥4, CRP ≥1 mg/dL); there were no recurrences observed among patients on continuous rilonacept therapy without interruption.

These data illustrate that recurrent pericarditis is a chronic disease often lasting for years, necessitating prolonged treatment. The current paradigm of repetitive cycling of nonspecific oral therapies (including corticosteroids) while attempting to balance duration‐dependent drug toxicities versus pericarditis recurrence risk may be clinically undesirable. Effective, targeted monotherapy with rilonacept, as evidenced by RHAPSODY, provides a favorable long‐term benefit–risk profile and advances the treatment paradigm to support consistent continued therapy throughout disease duration without interruption, lowering recurrence risk while on treatment.

As previously summarized, rilonacept treatment was associated with a marked reduction in pericarditis recurrence during the 18 months up to the decision milestone. While 3 investigator‐assessed recurrences were observed during this period, these reports did not meet formal RHAPSODY event adjudication criteria (ie, only symptoms, absence of CRP elevation). Empiric addition of prophylactic colchicine before the 18‐month decision milestone was minimal (5/74 patients) and was not considered a confounder; thus, the observed suppression of pericarditis recurrence while on treatment appears attributable primarily to rilonacept monotherapy.

Continued rilonacept treatment beyond 18 months resulted in continued treatment response, with a 98% reduction in risk of pericarditis recurrence in the nonrandomized comparison of continued rilonacept treatment beyond the 18‐month decision milestone versus suspension of rilonacept treatment for off‐treatment observation. The single recurrence in the rilonacept continuation arm was associated with an intentional 4‐week treatment interruption for elective surgery, mirroring the 2 recurrences in the rilonacept arm in the randomized‐withdrawal period, similarly associated with temporary interruptions of 1 to 3 weeks. The International Registry of Anakinra for Pericarditis also showed that longer treatment durations reduced recurrence risk.^11^

Conversely, even after 18 months of rilonacept treatment and despite the absence of pericarditis signs and symptoms (ie, absence of pain, CRP elevation, pericardial rub, ECG changes, or pericardial effusion) and the presence of only none/trace LGE in CMRs while on therapy, suspension of rilonacept treatment in these patients yielded a 75% (6/8) recurrence rate, likely due to an unmasking of the still‐persistent underlying autoinflammatory processes that had been previously quenched by the interleukin‐1‐trap mechanism of rilonacept. While LGE of the pericardium during a recurrence can inform prospective management,^12^ in this study, absence of LGE while on treatment did not predict reduced recurrence risk upon subsequent suspension of therapy.

Pericarditis recurrences occurred at 9 and 12 weeks after rilonacept treatment cessation during the randomized‐withdrawal and long‐term extension periods, respectively. This event timing parallels the expected gradual 5‐ to 8‐week fall in rilonacept plasma concentration (5 times 7‐day half‐life).^9^ Accordingly, among patients with off‐treatment recurrences in the randomized‐withdrawal period (ie, placebo randomization), patient‐reported outcomes (eg, pain, symptoms, and quality‐of‐life scores) showed incremental rather than abrupt worsening in the weeks leading to recurrence, demonstrating predictability of recurrence symptoms in most patients and facilitating timely reinitiation of rilonacept.^13^ Rilonacept reinitiation resolved acute pericarditis recurrences, including in 4 cases where attempted combination NSAID/colchicine rescue had failed. Similarly, in a patient registry of once‐daily anakinra over 9.2 months, extending the 6‐month treatment period with an additional 3 months of tapering reduced recurrences, although the overall longer total treatment duration (ie, including the tapering period) may have contributed to reduced recurrence rates.^11^

Five additional recurrences (meeting RHAPSODY event adjudication criteria) were recorded among 37 patients during the 6‐week safety follow‐up period after last on‐study dose of rilonacept. It is important to note, however, that the duration of follow up observation (6 weeks) was less than the time to event windows of the randomized withdrawal period (8.6 weeks) and in the long‐term extension suspension/observation period (13 weeks); potential events occurring after the 6‐week safety follow‐up period had ended would have been outside of the observational gaze of the trial, so these event counts may be an underestimate.

Collectively, these findings provide strong confirmatory evidence of interleukin‐1 being an important mediator in this disease, which is characterized by multiple painful recurrences and elevated CRP. The data also confirm that long‐term targeted interleukin‐1 pathway inhibition has clinical relevance and may offer optimal disease management to reduce multiple recurrences in patients failing first‐line oral therapies.

Rilonacept was generally well tolerated over the median 2‐year (maximum 3‐year) treatment period. Adverse events were consistent with the pivotal trial and prior rilonacept experience in other diseases. Upper respiratory tract infections were most common (16%), similar to the randomized‐withdrawal period (23%). Injection‐site reactions were notably lower in the long‐term extension versus the randomized‐withdrawal period (7% and 34%, respectively), possibly due to either accumulated tolerance or increased patient familiarity with injections.

Some patients discontinued rilonacept treatment because of intercurrent surgery or COVID‐19. The data suggest that interruption of rilonacept may increase pericarditis recurrence risk, adding to management challenges. It has been proposed that interleukin‐1 antagonists may be safely used perioperatively.^14^, ^15^ Similarly, it has been proposed to not discontinue interleukin‐1 antagonists during COVID‐19 infection.^1^, ^16^

Potential limitations of the randomized withdrawal design have been discussed previously.^4^ While the relatively small number of patients is a limitation, the open‐label design of the long‐term extension allowed for rilonacept treatment in all 74 patients—more than double the number of patients in the randomized‐withdrawal period rilonacept cohort (n=30) and over a 2‐ to 3‐fold longer period, with 52 of 74 patients reaching the 18‐month decision milestone. Despite the limitations of sample size in this rare disease study, the efficacy findings were robust and clinically significant. Although the open‐label design and the nonrandomized allocation of interventions are limitations, the nonrandomized subgroup of patients who voluntarily suspended rilonacept after the 18‐month decision milestone with observation effectively served as a small nontreated control group, who experienced a high recurrence event rate and demonstrated results that were consistent with the arm in the main study of patients who were randomized to placebo.

Another consideration was the fact that recurrences during the long‐term extension were investigator‐assessed and were not externally adjudicated; while this different approach in the long‐term extension was intended to more closely mirror clinical practice, some cases reported as recurrences did not meet formal RHAPSODY randomized‐withdrawal recurrence criteria, in which a CRP elevation >1 mg/dL was required in addition to the report of increased pain. As a result, 3 patients during continuous long‐term rilonacept therapy who reported only pain symptoms were classified by their investigator as having had “recurrences,” leading to a potential overestimate of the annualized incidence of recurrence while on continuous treatment. By contrast, all events associated with treatment interruption after suspension or discontinuation of rilonacept in the long‐term extension met canonical RHAPSODY event adjudication criteria, with larger‐magnitude pain NRS scores being accompanied by large CRP elevations, supporting the interleukin‐1 hypothesis. The characteristics in these events are consistent with the centrally adjudicated recurrence events in the randomized‐withdrawal period, in which CRP was elevated to >1 mg/dL in every case. Importantly throughout the entire base study and long‐term extension, there were no frank pericarditis recurrences with CRP elevation in patients on rilonacept in the absence of a treatment interruption.

Recurrent pericarditis is a chronic disease with a median duration of 3 years and significant interpatient disease duration variability, especially considering those with multiple recurrences may experience up to 5 years or 8 to 10 years of duration.^2^, ^3^, ^17^ Recurrence risk increases with each successive episode,^2^ and long‐term treatment, for years or indefinitely, may be necessary in some cases.^3^ The sustained effectiveness of rilonacept in clinical practice may obviate the need for initiating corticosteroids after failure of NSAIDs/colchicine in patients with multiple recurrences, mitigating the currently observed pattern of recurrences caused by therapy‐cycling secondary to safety concerns with prolonged use of current oral therapies, corticosteroids in particular, during the long disease course.

Determination of appropriate treatment duration when evaluating patients who are asymptomatic while on treatment remains a clinical challenge as no standardized biomarker currently exists which identifies a low risk of recurrence after treatment cessation. The potential utility of CMR in guiding clinical decision making may need further study, as the number of patients with CMRs at the 18‐month decision milestone in this study was limited. The accumulation of recurrences associated with premature treatment cessation worsens patient outcomes and burdens patient quality of life.^2^ Continued research into molecular, cellular, imaging, and genetic tools that clarify disease duration and off‐treatment recurrence risk is needed to guide decision making on therapeutic strategies and treatment duration.

## Conclusions

In conclusion, the RHAPSODY long‐term extension demonstrated that continued rilonacept treatment resulted in continued treatment response without accumulation of side effects, whereas treatment cessation in a small patient cohort was associated with a high risk of pericarditis recurrence even after 18 months of treatment. Consistent treatment for the full duration of the disease without interruption may be warranted for long‐term prevention in patients with recurrent pericarditis and disease characteristics similar to those studied in RHAPSODY. Larger studies would enable more precise generalization of these findings to the broader real‐world recurrent pericarditis patient population.

## Sources of Funding

This study was funded by Kiniksa Pharmaceuticals.

## Disclosures

Massimo Imazio is on the Kiniksa Pharmaceuticals scientific advisory board. Dr Imazio had full access to all the data in the study and takes responsibility for its integrity and the data analysis. Allan L. Klein reports a Kiniksa Pharmaceuticals research grant and scientific advisory board, Cardiol Therapeutics research grant and scientific advisory board, Pfizer Scientific Advisory Board, consultant to Kiniksa Pharmaceuticals. Antonio Abbate is a consultant and speaker for Kiniksa Pharmaceuticals. Antonio Brucato's institution received funding from Kiniksa Pharmaceuticals as an investigative site; unrestricted research grant from SOBI and ACARPIA; travel and accommodation for advisory committee from SOBI and Kiniksa. Paul C. Cremer reports grants and personal fees from Kiniksa Pharmaceuticals; grants from Novartis Pharmaceuticals; and personal fees from SOBI Pharmaceuticals outside the submitted work. Antonella Insalaco reports travel and accommodation for advisory committee from SOBI. Martin M. LeWinter reports consulting fees and grants from Kiniksa Pharmaceuticals. Basil S. Lewis reports consulting fees from Janssen Pharmaceuticals, Idorsia, CSL‐Behring (all outside the current work). Sushil A. Luis is a consultant for Kiniksa Pharmaceuticals and Medtronic. Stephen J. Nicholls reports grant support and consulting fees from AstraZeneca, Eli Lilly, Anthera Pharmaceuticals, Resverlogix, Sanofi Regeneron Pharmaceuticals, and Esperion Therapeutics; consulting fees from Akcea Therapeutics, Omthera Pharmaceuticals, Merck, Takeda Pharmaceutical, CSL Behring, and Boehringer Ingelheim; and grant support from Amgen, Novartis, Cerenis Therapeutics, the Medicines Company, LipoScience, Roche, and Infraredx. Paul Sutej is a speaker for Lilly. JoAnn Clair is an employee and shareholder of Kiniksa Pharmaceuticals. Indra Agarwal is an employee and shareholder of Kiniksa Pharmaceuticals. Sheldon Wang is an employee and shareholder of Kiniksa Pharmaceuticals. John F. Paolini is an employee and shareholder of Kiniksa Pharmaceuticals; inventor on patents/patent applications covering the use of rilonacept for the treatment of recurrent pericarditis. The remaining authors have no disclosures to report.

## Supporting information

Data S1.
Tables S1–S2.
