# Supplementary material for: Sustained Pericarditis Recurrence Risk Reduction With Long‐Term Rilonacept
Source: J Am Heart Assoc. 2024 Mar 12;13(6):e032516. doi: 10.1161/JAHA.123.032516 (PMC11010039; doi:10.1161/JAHA.123.032516)
Supplement: Supplementary file 1 — Data S1. Tables S1–S2. [file JAH3-13-e032516-s001.pdf]

SUPPLEMENTAL MATERIAL

RHAPSODY Investigators:

From the University of Virginia, Charlottesville, Virginia, USA [Antonio Abbate]; CardioVoyage, McKinney, Texas, USA [Wael Abo-Auda]; BI Research Center, Houston, Texas, USA [Asif Akhtar]; Chaim Sheba Medical Center, Ramat Gan, Israel [Michael Arad, Yishay Wasserstrum]; Galilee Medical Center, Nahariya, Israel [Shaul Atar]; Rochester General Hospital, Rochester, New York, USA [Bipul Baibhav]; Orion Medical, Pasadena, Texas, USA [Karan Bhalla]; ASST Fatebenefratelli Sacco - Ospedale Fatebenefratelli e Oftalmico, Milan, Italy [Antonio Brucato]; Vanderbilt University Medical Center, Nashville, Tennessee, USA [Sean Collins]; Core Research Group, Milton, Queensland, Australia [David Colquhoun]; Cleveland Clinic, Cleveland, Ohio, USA [Paul Cremer, Allan Klein]; HeartCare Partners Clinical Research Unit, Milton, Queensland, Australia [David Cross]; Fiona Stanley Hospital, Murdoch, Western Australia, Australia [Girish Dwivedi]; Rabin Medical Center – PPDS, Petach Tikva, Israel [Alon Eisen]; HaEmek Medical Center, Afula, Israel [Nahum Freedberg]; Assaf Harofe Medical Center, Tzrifin, Israel [Shmuel Fuchs]; Loretto Hospital, Chicago, Illinois, USA [Eliyazar Gaddam]; Istituto G Gaslini Ospedale Pediatrico IRCCS, Genova, Italy [Marco Gattorno]; Beth Israel Deaconess MC, Boston, Massachusetts, USA [Eli Gelfand]; Cardiology Consultants of Philadelphia, Yardley, Pennsylvania, USA [Paul Grena]; ZIV Medical Center, Zefat, Israel [Majdi Halabi]; University of Cincinnati, Cincinnati, Ohio, USA [David Harris]; Azienda Ospedaliero Città della Salute e della Scienza di Torino, Turin, Italy [Massimo Imazio]; Ospedale Pediatrico Bambino Gesù, Rome, Italy [Antonella Insalaco]; Angiocardiac Care of Texas PA, Houston, Texas, USA [Amin Karim]; Intermountain Healthcare, Murray, Utah, USA [Kirk Knowlton]; Stony Brook University School of Medicine, Stony Brook, New York, USA [Apostolos Kontzias]; Icahn School of Medicine at Mount Sinai, New York, New York, USA [Robert Kornberg]; Oklahoma City VA Medical Center – NAVREF, Oklahoma City, Oklahoma, USA [Faisal Latif]; Hadassah University Hospital Mount Scopus, Jerusalem, Israel [David Leibowitz]; University of Vermont Medical Center, Burlington, Vermont, USA [Martin LeWinter]; Minneapolis Heart Institute Foundation, Minneapolis, Minnesota, USA [David Lin]; GenesisCare – Cardiology Research, Doncaster, East Victoria, Australia [Pey Wen Lou]; Mayo Clinic, Rochester, Minnesota, USA [S. Allen Luis]; Monash Health, Monash Medical Centre, Clayton, Victoria, Australia [Stephen Nicholls]; Swedish Medical Center, Seattle, Washington, USA [John Petersen]; Seattle Children's Hospital, Seattle, Washington, USA [Michael Portman]; Royal Hobart Hospital, Hobart, Tasmania, Australia [Philip Roberts-Thomson]; Bnai Zion Medical Center, Haifa, Israel [Elad Schiff]; Cedars-Sinai Heart Institute, Los Angeles, California, USA [Robert Siegel]; The Queen Elizabeth Hospital, Woodville, South Australia, Australia [Michael Stokes]; Arthritis and Rheumatology of Georgia, Atlanta, Georgia, USA [Paul Sutej]; Cincinnati Children's Hospital Medical Center, Cincinnati, Ohio, USA [Samuel Wittekind]; Edith Wolfson Medical Center, Holon, Israel [Valentin Witzling]; Rambam Health Corporation, Haifa, Israel [Robert Zukermann].

Data S1.

Supplemental Methods

Patient Global Impression of Pericarditis Severity (PGIPS) and Physician Global Assessment of Pericarditis Activity (PGA-PA)

The PGIPS is a single-item measure of a person's impression of the overall severity of their pericarditis symptoms at the time the questionnaire is administered; impressions are rated on a 7-point scale ranging from absent (0, no recurrent pericarditis symptoms) to very severe (6, recurrent pericarditis symptoms that cannot be ignored and markedly limit daily activities). The PGA-PA is a single-item measure of an investigator's impression of a patient's overall pericarditis disease activity at the time the assessment is completed; impressions are rated on a 7-point scale ranging from absent to very severe. PGIPS administrations and PGA-PA assessments were planned every 12 weeks during participation in the long-term extension period.

Annualized Recurrence Rate Determinations

The annualized recurrence rate was determined as the number of recurrences in long-term extension periods for all patients, divided by the sum of patient-years in long-term extension periods for all patients. Similarly, the annualized recurrence rate was calculated in the long-term extension period, up to the 18-month decision milestone as well as after this milestone. For patients who remained on study after the milestone but were off treatment (observation only), patient-years were calculated as treatment, minimum (end-of-study date, first-dose date after observation –1) – long-term extension 18-month

disposition date +1. For patients who continued treatment, patient-years were calculated as end-of-study date – long-term extension 18-month disposition date +1. The 95% confidence interval was calculated with an exact method with Poisson distribution.

Supplemental Results

Patients' Recurrence Narratives

Investigator-Assessed Pericarditis Recurrences Before 18-Month Decision Milestone

Three investigator-assessed pericarditis recurrences were experienced before the 18-month decision milestone.

(1) This 29-year-old female with a 1.2-year prior history of "idiopathic" RP enrolled in RHAPSODY during her 2nd pericarditis recurrence. Because the randomized withdrawal (RW) period had closed prior to potential randomization, the patient went directly from the Run-In period to the Long-term extension (LTE). At Week 52, at the LTE end-of-treatment visit (done remotely) the investigator recorded a recurrence. The NRS was 8, and CRP was 0.73 mg/dL. No ECG changes were observed, no pericardial effusion was observed, and presence of pericardial rub was not assessed. There had been no interruption of rilonacept treatment prior to the episode, and the patient transitioned to commercial rilonacept. No oral rescue therapy was added.

(2) This 37-year-old female with a 0.7-year prior history of "idiopathic" RP enrolled in RHAPSODY during her 6th pericarditis recurrence. A CMR obtained at Run-In (RI) baseline rated LGE as "mild." During the RW period, she was randomized to placebo and experienced a recurrence; bailout rilonacept was administered. At approximately Week 26 in the LTE and prior to the 18-month decision milestone, the subject had an investigator-assessed recurrence. The NRS was 7, and CRP was 0.28 mg/dL. No ECG changes were observed, a new effusion was observed, and there was no pericardial rub. There had been no interruption of rilonacept treatment prior to the episode. Treatment with NSAID was added for six weeks, and rilonacept was continued. The subject completed the LTE without further recurrences.

(3) This 59-year-old female with a 0.3-year prior history of "idiopathic" RP enrolled in RHAPSODY during her 2nd pericarditis recurrence. A CMR obtained at RI baseline rated LGE as "severe." During the RW period, she was randomized to placebo and experienced a recurrence; bailout rilonacept was administered. Between Weeks 12 and 24 in the LTE and prior to the 18-month decision milestone, the subject had an investigator-assessed recurrence. The NRS was 5, and CRP was 0.2 mg/dL. There were no ECG changes, pericardial effusion, nor pericardial rub. There had been no interruption of rilonacept treatment prior to the episode. Treatment with NSAID and colchicine was added for 3 weeks and for 2 days, respectively, and rilonacept was continued. The subject completed the LTE without further recurrences.

Investigator-Assessed Pericarditis Recurrences After 18-Month Decision Milestone

(a) Pericarditis Recurrence While Continuing on Rilonacept

There was one investigator-assessed recurrence among the 33 patients who continued on rilonacept treatment beyond the 18-month decision milestone.

(1) This 29-year-old male with a 0.4-year prior history of “idiopathic” RP enrolled in RHAPSODY during his 3rd recurrence. During the RW period, he was randomized to placebo and experienced a recurrence; bailout rilonacept was administered. An investigator-assessed pericarditis recurrence occurred 23.4 weeks into the LTE. Of note, this subject had been instructed to interrupt rilonacept treatment 2 weeks in advance of an elective aortic valve replacement surgery. A partial pericardiectomy was also performed. Approximately 10 days after the cardiac surgery and 4.5 weeks following the interruption of rilonacept, the subject returned to clinic complaining of chest pain and having taken a single dose of prednisone 30 mg. NRS was 8, and CRP was 7.5 mg/dL. No ECG changes were observed, no pericardial effusion was observed, and there was no pericardial rub. The patient was instructed to stop prednisone and was started on colchicine 0.5 mg bid. Rilonacept was reinitiated with a loading dose 26 days after the recurrence. The subject remained on rilonacept and colchicine 0.5 mg qd for the remainder of the LTE (10 weeks) with no subsequent recurrences and transitioned to post-study rilonacept.

(b) Pericarditis Recurrence After Suspension of Rilonacept

Among the 8 patients who suspended rilonacept treatment at the 18-month decision milestone and remained on-study for observation, 6 patients (75%) subsequently had an investigator-assessed recurrence. The following provides additional details regarding these recurrences and the medical management that resulted. Select clinical measures that were obtained at the 18-month assessment are also provided.

(1) This 63-year-old female with a 0.5-year history of “idiopathic” recurrent pericarditis enrolled in RHAPSODY during her 3rd recurrence. During the RW period, she was randomized to placebo and had a recurrence. At the 18-month decision milestone (18 months from the RW period recurrence), the CRP level was 0.2 mg/dL, the ECG was normal, and no effusion nor pericardial rub was observed. A CMR obtained 12 days prior to the assessment rated LGE as “none.” The decision to suspend rilonacept treatment was made. Paracetamol was started 3.5 weeks later for chest pain, and NSAID (ibuprofen, 600 mg bid) and colchicine (0.5 mg every 2 days) were started 2 days later. The subject continued to experience increased pain, and an investigator-assessed pericarditis recurrence was recorded approximately 4.5 weeks after the last (18 month) rilonacept dose. The NRS was 8, and CRP was 4.9 mg/dL. There were ECG changes, a new effusion, and a pericardial rub. Rilonacept was reinitiated as bailout (with a loading dose) on the day in which recurrence was assessed, and NSAID and colchicine were rapidly tapered over 7 days and discontinued. The subject continued rilonacept monotherapy through the end of the study with no additional recurrences.

Rilonacept was discontinued at the end of the LTE (Week 103), and anakinra was started prophylactically 3 weeks into the post-treatment safety follow-up period.

(2) This 49-year-old male with a 1.5-year history of “idiopathic” recurrent pericarditis enrolled in RHAPSODY during his 2nd recurrence. During the RW period, he was randomized to placebo and did not have a recurrence. At the 18-month decision milestone (18 months from the qualifying event), the CRP level was 0.2 mg/dL, the ECG was normal, and no effusion nor pericardial rub was observed. A CMR obtained on the day of the assessment rated LGE as “none.” The decision to suspend rilonacept treatment was made. An investigator-assessed pericarditis recurrence was recorded approximately 11 weeks after the last (18 month) rilonacept dose. NRS was 7, and CRP was 1.6 mg/dL. There were no ECG changes, and no effusion nor pericardial rub was observed. Initial treatment for the recurrence was paracetamol and tramadol for 1 week, followed by an NSAID for 1 week without relief. Rilonacept was reinitiated as bailout (without loading dose) five weeks post recurrence. The subject continued rilonacept monotherapy through the end of the LTE (31 weeks) with no additional recurrences.

(3) This 28-year-old female with a 1.1-year prior history of “idiopathic” recurrent pericarditis enrolled in RHAPSODY during her 3rd recurrence. During the RW period, she was randomized to rilonacept and did not have a recurrence. At the 18-month decision milestone (18 months from the qualifying event), the CRP level was 0.4 mg/dL, the ECG was normal, and no effusion nor pericardial rub

was observed. The decision to suspend rilonacept treatment was made, and 1 week later the patient reported increasing pain. NSAID and colchicine were started at a clinic visit approximately 2 weeks later. An investigator-assessed pericarditis recurrence was recorded 9 days later, approximately 4.5 weeks after the last (18 month) rilonacept dose. The NRS was 8, and CRP was 3.4 mg/dL. The ECG was normal, and no effusion nor pericardial rub was observed. Rilonacept was reinitiated as bailout (with a loading dose). NSAID was stopped when rilonacept was started, and colchicine was tapered within 6 weeks. The patient continued rilonacept monotherapy through the end of the LTE with no additional recurrences.

(4) This 42-year-old female subject with a 2.9-year prior history of “idiopathic” recurrent pericarditis enrolled in RHAPSODY during her 3rd recurrence. A CMR obtained at RI baseline rated LGE as “severe.” During the RW period, she was randomized to rilonacept and did not have a recurrence. At the 18-month decision milestone (18 months from the qualifying event), the CRP level was 0.08 mg/dL, the ECG was normal, and no effusion nor pericardial rub was observed. A CMR obtained on the day of the assessment rated LGE as “trace” (less than 50% circumferential extent at base, mid, and apex). The decision to suspend rilonacept treatment was made, and an investigator-assessed pericarditis recurrence was recorded approximately 15 weeks after the last (18 month) rilonacept dose. The NRS was not available, and CRP was 1.2 mg/dL. ECG, echocardiogram, and pericardial exam were not available. Treatment for the

recurrence included ibuprofen (600 mg, tid) for approximately 2 weeks. The subject continued through the end of the LTE (approximately 19 weeks) with no additional recurrences.

(5) This 51-year-old female with a 3.3-year history of “idiopathic” recurrent pericarditis enrolled in RHAPSODY during her 4th recurrence. A CMR obtained at RI baseline rated LGE as “mild.” During the RW period, she was randomized to rilonacept and did not have a recurrence. At the 18-month decision milestone (18 months from the qualifying event), the CRP level was 0.2 mg/dL, the ECG was normal, and no effusion nor pericardial rub was observed. A CMR obtained on the day of the assessment rated LGE as “none.” The decision to suspend rilonacept treatment was made, and 1 week later colchicine (0.6 mg) was started prophylactically in the absence of reported chest pain; 11 weeks later, at approximately 12 weeks after the last (18 month) rilonacept dose an investigator-assessed pericarditis recurrence was recorded. The NRS was 8, and CRP was 1.6 mg/dL. The ECG was normal, and no effusion was present. There was no pericardial rub. Treatment included continuation of colchicine, and NSAID was added 1 day after the recurrence. Because of persistent pain, rilonacept was reinitiated as bailout (with loading dose) 1 week after the recurrence on top of the NSAID and colchicine, and the patient remained on rilonacept without recurrence until the end of the LTE (13 weeks later), when the subject was switched to commercial rilonacept.

(6) This 63-year-old male with a 3.2-year history of “idiopathic” recurrent pericarditis enrolled in RHAPSODY during his 4th recurrence. A CMR obtained at RI baseline rated LGE as “moderate.” During the RW period, he was randomized to rilonacept and did not have a recurrence. At the 18-month decision milestone (18 months from the qualifying event), the CRP level was 0.07 mg/dL, the ECG was normal, and no effusion or pericardial rub was observed. A CMR obtained on the day of the assessment rated LGE as “trace” (less than 50% circumferential extent at base, mid, and apex). The decision to suspend rilonacept treatment was made, and 4 weeks later colchicine (0.6 mg) was started prophylactically in the absence of reported chest pain. An investigator-assessed pericarditis recurrence was recorded 9 weeks later, approximately 13 weeks after the last (18 month) rilonacept dose. Colchicine was continued, and aspirin (650 mg) was added. NRS was 8, and CRP was 8.6 mg/dL at an RP-event assessment performed 4 weeks after the date of recurrence onset. The ECG was normal, no effusion was observed, and there was no pericardial rub. Rilonacept was reinitiated as bailout (with loading dose) 4 weeks after the recurrence. The NSAID and colchicine were stopped 3 weeks and 14 weeks, respectively, after rilonacept was reinitiated. The subject continued on rilonacept without recurrence until the end of the LTE (16 weeks later) and then switched to commercial rilonacept.

(c) Absence of Pericarditis Recurrence After Suspension of Rilonacept

Among the 8 subjects who suspended rilonacept treatment at the 18-month milestone and remained on-study for observation, 2 subjects (25%) had no recurrence.

(1) This 26-year-old male with a 1.4-year history of “idiopathic” recurrent pericarditis enrolled in RHAPSODY during his 2nd recurrence. A CMR obtained at RI baseline rated LGE as “severe.” During the RW period, he was randomized to rilonacept and did not have a recurrence. At the 18-month decision milestone (18 months from the qualifying event), the CRP level was 0.05 mg/dL, the ECG was normal, and no effusion or pericardial rub was observed. A CMR obtained on the day of the assessment rated LGE as “trace” (less than 50% circumferential extent at base, mid, and apex). The decision to suspend rilonacept treatment was made. Colchicine was initiated prophylactically in the absence of reported chest pain 1 week after the last dose of rilonacept, and no recurrence was reported through the end of the LTE.

(2) This 44-year-old male with a 1.4-year history of post-cardiac injury recurrent pericarditis enrolled in RHAPSODY during his 3rd recurrence. A CMR obtained at RI baseline rated LGE as “mild.” During the RW period, he was randomized to rilonacept and did not have a recurrence. At the 18-month decision milestone (18 months from the qualifying event), the CRP level was 0.03 mg/dL, the ECG was normal, and no effusion or pericardial rub was observed. A CMR obtained on the day of the assessment rated LGE as “none”. The decision to suspend rilonacept

treatment was made, no recurrence was reported through the end of the LTE. No prophylactic medicine was initiated.

Investigator-Assessed Pericarditis Recurrences Observed During 6-Week Safety Follow-up Period (After Rilonacept Study Treatment Cessation/Discontinuation)

At the end of the LTE treatment period, all non-US patients who were still on rilonacept study drug (n=25) were returned to standard of care for RP and followed for an additional six-week safety follow-up period. During this post-treatment follow-up, there were five additional investigator-assessed pericarditis recurrences in these 25 subjects. There were no recurrences in the 10 patients from the US who discontinued rilonacept treatment at commercialization nor in the 11 patients who discontinued rilonacept treatment at the 18-month decision milestone.

(1) This 57-year-old male with a 0.9-year history of “idiopathic” recurrent pericarditis enrolled in RHAPSODY during his 3rd recurrence. During the RW period, he was randomized to placebo and did not have a recurrence. He started rilonacept without loading dose at the beginning of the LTE. At the 18-month decision milestone (18 months from qualifying event) he continued on rilonacept. He received 117 weeks of therapy in total. The recurrence was recorded approximately 3 weeks after the last rilonacept dose in the LTE safety follow-up period. The patient complained of pericarditic chest pain and fever. NRS was 6, and CRP was 2.0 mg/dL. ECG showed ST elevation in precordial leads. No

effusion was observed. Pericardial rub was not present. The subject received treatment with NSAID and colchicine.

(2) This 23-year-old male subject with an 11.2-year history of “idiopathic” recurrent pericarditis enrolled in RHAPSODY during his 10th recurrence. During the RW period, he was randomized to placebo and had a recurrence. He received bailout rilonacept. At the 18-month decision milestone he continued on rilonacept. A CMR obtained on the day of the assessment rated LGE as “none.” He received 129 weeks of therapy in total. The recurrence during the six-week safety follow-up period was recorded approximately six weeks after the last rilonacept dose in the LTE. NSAID was started 3 days prior to the recurrence for pain. The NRS was 7 and CRP was 2.5 mg/dL. ECG was not performed, echo showed no effusion, and pericardial rub was not present. Treatment for the recurrence included continued NSAID and addition of anakinra, which was ongoing at the end of the safety follow-up period.

(3) This 16-year-old male subject with a 1.8-year history of “idiopathic” recurrent pericarditis enrolled in RHAPSODY during his 3rd recurrence. During the RW period, he was randomized to placebo and had a recurrence. He received bailout rilonacept. At the 18-month decision milestone he continued on rilonacept. A CMR obtained on the day of the assessment rated LGE as “none”. He received 123 weeks of therapy in total. The recurrence was recorded approximately six weeks after the last rilonacept dose in the LTE. The NRS was 8 and CRP was

1.9 mg/dL. No pericarditis-related ECG change, echo showed no effusion, and pericardial rub was not present. Treatment included anakinra starting on the day of the recurrence, which was ongoing at the end of the safety follow-up period.

(4) This 27-year-old male subject with a 1.2-year history of “idiopathic” recurrent pericarditis enrolled in RHAPSODY during his 2nd recurrence. The patient entered the LTE directly from the Run-In period. At the 18-month decision milestone he continued on rilonacept. He received 117 weeks of therapy in total. The recurrence was recorded approximately six weeks after the last rilonacept dose in the LTE. The NRS was 7, and CRP was 4.2 mg/dL (peak CRP 28 mg/dL). ECG showed changes typical for acute pericarditis including ST elevation and PR depression. Echo showed mild effusion with fibrin. No pericardial rub was present. The subject was prescribed NSAIDS, colchicine, and eventually prednisone 40 mg, which was ongoing at the end of the safety follow-up period.

(5) This 62-year-old male subject with a 0.4-year history of recurrent pericarditis from post-pericardiotomy syndrome enrolled in RHAPSODY during his fourth recurrence. During the RW period, he was randomized to placebo and had a recurrence. He received bailout rilonacept. At the 18-month decision milestone, he continued on rilonacept. At 59 weeks post entry into the LTE (4.5 months after the 18-month decision milestone), he was hospitalized due to acute endocarditis, leading to discontinuation of rilonacept, having received 109 weeks of therapy in

total. One week following the acute endocarditis event, he experienced a non-ST elevation myocardial infarction (NSTEMI), prolonging his hospitalization and leading to coronary artery bypass graft surgery and redo aortic valve replacement surgery. Approximately 6 weeks after the last dose of rilonacept, he experienced an acute pericarditis event (CRP, 8.7 mg/dL). He was treated with colchicine, which was ongoing at the end of the safety follow-up period.

RHAPSODY Run-In and Randomized-Withdrawal Period Data Updates with Final Database Lock and Closing of the Long-Term Extension

This dataset update, which came about as a result of the final database lock and closing of the Long-Term Extension (LTE) of the trial, did not impact the primary endpoint (reduction in risk of adjudicated pericarditis recurrence) nor the conclusions drawn from any of the secondary endpoints of the primary trial, and resulted in certain small numerical differences in a few of the data elements reported in the original RHAPSODY manuscript and secondary analysis manuscript.[4,13]

First, because a single electronic data collection (EDC) database was used for all periods of the trial (Screening, Run-In (RI), Randomized-Withdrawal (RW), and LTE), during the routine verification of data entries pertaining to the LTE period, some of the data entry updates contained values that were pertinent to the RI and RW period analytes as well. As a result, when the data tables were re-run per routine after final database lock, several small numeric differences were identified in certain individual

point estimates for some RI and RW period analytes when compared to the analysis conducted at the end of the RW period.

Second, eDiaries were used throughout all periods of the trial to collect patient-reported outcomes (ePRO) data, for example, the pericardial pain NRS score. Overall, there was high rate of completion of eDiaries, with a 95% completion rate in the trial, and a low degree of missing data (0.2%) in the original analysis of the Run In (RI)/Randomized Withdrawal (RW) periods. The primary analysis used prespecified rules that had been discussed with the FDA to impute specific data values for any missing data points. Recently, during the process of closing the LTE, the eDiaries used by patients in the trial were returned to the vendor, and a small number of previously-missing individual ePRO data entries from the RI and RW periods became available. In all, 68 new pericardial pain NRS individual data entries (out of a total of 16,905) were identified; we replaced those specific previously-imputed datapoints with the actual data entries into the final dataset, and the analyses were re-run.

The data updates are reported below. Individual subgroup analyses are available upon request.

Median time to rilonacept monotherapy (Run-In Period)

After final database lock, due to adjustments to the start and stop dates of some concomitant medication entries, the time to rilonacept monotherapy in the RI period (7.9 weeks) was recalculated to be 7.3 weeks (95% CI, 7.0 to 8.1) and the number of

patients who achieved monotherapy (74 patients) was recalculated to be 73 patients. Additionally, in patients with 2-4 episodes per year and in patients with disease duration of 1-2 years, the median time to rilonacept monotherapy endpoint in the RI period changed from 8.3 to 8.0 weeks and from 8.0 to 7.6 weeks, respectively.

Mean numerical rating scale scores for pain over the 12-week run-in period

Mean numerical rating scores (NRS) were updated at week 3 from 1.04 to 1.03, week 8 from 0.61 to 0.60 and week 12 from 0.46 to 0.45. Final Run-In period NRS data are reported in Table S1.

Randomized-withdrawal trial endpoint of days with no or minimal pain

The least-squares mean percentage of days with no or minimal pericarditis pain (secondary efficacy endpoint) was updated from 97.7 (± 7.5) days to 98.6 (± 7.6) days in the active arm and from 45.9 (± 7.2) days to 47.4 (± 7.3) days in the placebo arm. Table S2 reports the final updated data.

Table S1. Final mean numerical rating scores (NRS) during Run-In period.

Run-in Visit	Baseline	Week 1	Week 2	Week 3	Week 4	Week 5	Week 6	Week 7	Week 8	Week 9	Week 10	Week 11	Week 12
**No. of patients**	84	84	84	84	83	84	83	83	83	81	82	82	78
**Mean NRS value**	4.50	1.60	1.43	1.03	1.13	0.86	0.78	0.68	0.60	0.76	0.69	0.53	0.45

Table S2. Randomized-withdrawal Days with no or minimal pain*.

	Rilonacept (N=30)	Placebo (N=31)	Hazard Ratio or Difference (95% CI)	P Value
**No. of patients included in analysis**	21	20	51.8 (35.3-68.4)	<0.001
**Least-squares mean percentage[†]**	98.6 ± 7.6	47.4. ± 7.3		

*No or minimal pain was defined as a nonmissing daily pericarditis pain score of no more than 2, as assessed on the numerical rating scale. The percentage of days with no or minimal pain during the first 16 weeks was calculated for each patient with the use of 112 days (i.e., 16 × 7 days) as the denominator. Days with missing values in the pain diary were counted as 0 days with no or minimal pain, as were days with use of an oral rescue therapy or glucocorticoid. If bailout rilonacept was used, each administration (loading dose or not) was counted as 7 days during which “no or minimal pain” could not be noted.

[†]The least-squares mean difference was calculated for the rilonacept group minus the placebo group. The two-sided P value for this analysis was calculated by an analysis of covariance with trial group, randomization strata, and the category for the weekly mean numerical rating scale score (≤2 vs. >2) at baseline of the run-in period as covariates.
